# School-Level Perspectives of the Ontario Student Nutrition Program

**DOI:** 10.3390/children9020177

**Published:** 2022-02-01

**Authors:** Mariam R. Ismail, Jason A. Gilliland, June I. Matthews, Danielle S. Battram

**Affiliations:** 1 School of Health and Rehabilitation Sciences, Western University, London, ON N6A 3K7, Canada; mismail8@uwo.ca; 2 Department of Epidemiology and Biostatistics, Western University, London, ON N6A 5C1, Canada; jgillila@uwo.ca; 3 Schulich School of Medicine and Dentistry, Western University, London, ON N6A 5C1, Canada; 4 Department of Geography and Environment, Western University, London, ON N6A 5C2, Canada; 5 Department of Paediatrics, School of Health Studies, Western University, London, ON N6A 3K7, Canada; 6 Human Environments Analysis Laboratory, Western University, London, ON N6A 3K7, Canada; 7 Children’s Health Research Institute, Lawson Health Research Institute, London, ON N6C 2V5, Canada; 8 School of Food and Nutritional Sciences, Brescia University College, London, ON N6G 1H2, Canada; jmatth22@uwo.ca; 9 Schulich Interfaculty Program in Public Health, Western University, London, ON N6A 3K7, Canada

**Keywords:** school food program, children, implementation, volunteer-led

## Abstract

The purpose of this study was to assess the implementation of a school snack program for children in elementary schools. School-level program volunteers’ experiences were explored using semi-structured interviews. Fieldnotes were taken during on-site school visits. Quantitative data were collected through a General Information Form and Weekly Logbooks. Seven elementary schools in Southwestern Ontario were invited and agreed to participate. Interviews (*n* = 27) revealed that volunteers valued the program for its universality, the excitement it created, the opportunity for students to try new foods, and the social interactions that it generated. Challenges included the burden on snack volunteers to plan, procure, and prepare foods; the competition the program posed for school priorities; limited funding; and a lack of clear purpose for the program. Suggestions for improvement included providing adequate and sustained resources and an integration of the program into the curriculum. Data obtained from 15 on-site visits, 7 General Information Forms, and 59 (out of a total of 70) Weekly Logbooks confirmed the data obtained from interviews. This research provides insights into the challenges of volunteer-led school snack programs in Canada and may guide policy makers, practitioners, and researchers in the development of a universal, nationally funded school food program.

## 1. Introduction

School food programs (SFP) have been identified as promising strategies for fostering children’s healthy development [1]. In Canada [2,3] and internationally [4], SFP have shown positive impacts on children’s intake of healthy foods (e.g., fruit and vegetables (FV)) and several psychosocial factors (e.g., attitudes, knowledge, and self-efficacy) known to mediate healthy eating behavior [5]. SFP also have the potential to improve children’s food literacy [6], reduce social disparities in FV consumption [7], strengthen the local food system [8], and may provide an effective response to food insecurity [9].

Although different types of SFP have been implemented, Canada remains one of the few industrialized countries without a universal, nationally funded SFP [10]. The responsibility for existing SFP falls mainly on provincial and territorial governments, often in collaboration with local organizations and businesses whose funding is usually limited due to competing priorities (e.g., health and education) [11]. Furthermore, these programs are often implemented by school staff and/or volunteers who are responsible for all the planning, food procurement, and preparation, leading to many diversified and inconsistent approaches to SFP processes and practices [12].

In Southwestern Ontario, the Ontario Student Nutrition Program (OSNP) is housed within the Windsor-Essex site of the Victorian Order of Nurses (VON). VON is 1 of 14 lead agencies in the provincial Student Nutrition Ontario program that administers provincial grant funds to support the development and implementation of SFP across the province [13]. The OSNP facilitates individual schools in the logistics of developing and implementing food programs with the overall purpose of providing universal access to healthy foods to thousands of school-aged children. Under the ‘usual practice’ model, schools receive funding on a per student basis and implement a SFP based on their unique capacities (e.g., number of volunteers and level of infrastructure). Ultimately, this determines the type of program administered (e.g., meal, snack, or breakfast) and the frequency and dose of food offerings. Schools are required to follow OSNP standards and procedures including (1) nutrition guidelines, such as offering a FV at each snack/meal; (2) spending guidelines (e.g., restrictions on funding to support only food purchases); and (3) the completion of administrative tasks (e.g., tracking foods provided and receipts). While this usual practice model allows schools the flexibility to implement a SFP that meets their individual needs, the practices involved across schools are inconsistent and not fully understood.

If a universal, nationally funded SFP is to be implemented in Canada, then a better understanding of the implementation practices and processes of existing SFP is clearly needed. To date, few SNP in Canada have undergone process evaluations [12,14,15]. The purpose of this study was to explore the feasibility and acceptability of the usual practice model of the OSNP in Southewestern Ontario from the perspective of school-level stakeholders. The findings may provide insights to guide policy makers, practitioners, and researchers in the development of a universal, nationally funded SFP in Canada.

## 2. Materials and Methods

### 2.1. Overview of Study Design

This research is part of a larger study that involved 60 elementary schools. Of these, 30 schools implemented the usual practice model of the OSNP while the remaining 30 schools received an enhanced intervention program that involved central food procurement and the delivery of foods to schools. The evaluation of the intervention arm of this study has been published elsewhere [15]. Although these schools implemented their SFPs over the entire school year (Sept to June), the current research was conducted over three 10-week phases of implementation: Phase I (Winter 2017) (*Ph1*); Phase II (Fall 2018) (*Ph2*); and Phase III (Winter 2018) (*Ph3*) to mirror the evaluation of the intervention arm of the larger study.

### 2.2. Participant Recruitment

To be eligible to participate in the study, schools were required to serve food 3–5 days a week. Of the 30 schools implementing the usual practice model of the OSNP, seven were invited to participate in this evaluation. All participating schools served children in full-day kindergarten through Grade 8, and schools in both urban and rural areas were selected to capture diversity in school characteristics. School size ranged from approximately 100 to 900 students. All personnel (e.g., administrative staff (AS), teachers (T), educational assistants (EA), and volunteers (V)) either directly involved with or aware of their school’s food program were invited to participate in the study. Recruitment occurred primarily by placing a recruitment flyer and the study’s Letter of Information in the staff lounge. Principals also made recruitment announcements on the day of the interviews. Any school-level participants who expressed interest in the study were interviewed. This study was approved on 29 November 2016 by the Western University Non-Medical Research Ethics Board (#108549) and the research and evaluation offices of the London District Catholic School Board and the Thames Valley District School Board. Written and/or verbal informed consent was obtained at the beginning of each interview.

### 2.3. Data Collection Methods

To gain a deep understanding of school-level staff and volunteer perspectives, we employed a descriptive, exploratory approach with the intention to advance knowledge translation between researchers, practitioners (stakeholders), policy makers, and the public and to provide suggestions for program improvement [16]. We also included general aspects of process evaluation to assess implementation practices (e.g., dose, reach, fidelity, barriers, and facilitators) [17].

Qualitative data were obtained through interviews and field notes from on-site visits. Interviews were conducted at the end of each phase of SFP implementation during school hours on dates determined in collaboration with the school principals. A semi-structured interview guide was adapted to reflect each participant’s involvement with the OSNP and to allow role-specific concepts to be explored (Table 1). On-site visits involved observing and making field notes on program implementation practices during each 10-week phase of the OSNP.

Quantitative data were derived from a General Information Form and Weekly Logbooks. Participants recorded data on general school and program characteristics (e.g., school enrollment and program type) on the General Information Form, which was administered once at the beginning of the program. Volunteers directly involved in food preparation recorded quality aspect measures (e.g., freshness of food items and degree of spoilage) in Weekly Logbooks.

### 2.4. Data Analysis

All interviews were audio recorded, transcribed verbatim by student volunteers, and verified by one of the researchers. Interviews were analyzed using inductive content analysis [18]. To increase reliability, transcripts were independently coded by three researchers, two of whom were experienced in qualitative research methods and analysis. Coding discrepancies were resolved through consensus until a common theme template was developed. Field note data were compiled and analyzed according to the theme template generated from the interviews. For quantitative logbook data, response categories (e.g., yes/no, poor to excellent) were calculated for each dimension using descriptive statistics, and organized using Microsoft Excel (Office 365, Microsoft Corp., Playa Vista, CA, USA, 2019).

Several strategies enhanced the trustworthiness of the data. The semi-structured interview guide was employed to achieve reliability and consistency in data collection. Member checking was conducted during interviews to confirm participants’ perspectives. An audit trail, including a reflexive journal, was kept as documentation of the decisions made during the analytical processes [19]. Validity was enhanced through the triangulation of data sources (e.g., field notes, interviews, and logbooks), a breadth of perspectives from a variety of school-level participants, a prolonged involvement of researchers with participants, and team data analysis [19].

## 3. Results

All seven invited schools that were implementing the usual practice model of the OSNP agreed to participate in this study (Table 2). In total, 27 interviews (24 in-person; 3 via telephone) were conducted (range: 45–60 min). Interview participants represented a variety of school roles: AS (*n* = 3); T (*n* = 10); EA (*n* = 6); and V (*n* = 8). In addition, 15 on-site visits were conducted (MRI). Each school was observed at least twice during their 10-week phase of implementation for approximately 2–3 h per visit. Each quotation is identified by participant role, participant number, and phase of implementation. Thus, ‘EA3_Ph2’ represents Educational Assistant, Participant #3, Phase 2. Seven General Information Forms (one per school) and 59 out of 70 Weekly Logbooks (response rate: 84.2%; range: 7–10 per school) were collected and analyzed.

### 3.1. Value of the Program

Most participants expressed appreciation for the OSNP and stated that it was a valuable and integral part of their school. Participants felt that the program supported and filled a need in their school community by feeding hungry children. Some participants also stated that the program showed children that they were cared for and provided support to parents and caregivers. In addition, most participants valued the universality of the program as it eliminated any potential stigma that some children may feel when accessing the program.

*“It’s a multi-pronged situation, that they’re feeding themselves, they’re getting the nutrition, and they know that somebody cares about them”*

EA1_Ph1

*“It gives students who might not necessarily come out and say they’re hungry a chance to get something to eat…there’s not so much stigma attached to it”*

EA6_Ph3

### 3.2. Successes of the Program

When asked about the successful aspects of snack programs, most participants focused on the benefits or impacts on their school communities. Frequently mentioned was the fact that the OSNP created excitement among children, with a few participants commenting that children expected or relied upon the program. This was confirmed by Weekly Logbook data from snack personnel, which suggested that children seemed to enjoy the snack 100% of the time (data not shown).

*“I get “thank yous”, I even get applauses sometimes, because the kids are excited; they’re happy”*

EA3_Ph2

*“There’s more bathroom visits I think than any other day of the week because they wanna know what I am up to”*

V8_Ph3

*“And they don’t have to worry about it, it’s not something special. They know they can count on it and it’s a comfort to them”*

EA4_Ph3

Most participants also mentioned that the snack program provided and exposed children to a variety of healthy foods that might not be offered to them at home. In turn, participants highlighted the impacts of this provision and exposure. Most participants stated that it encouraged children to try new items, helped them to focus on their learning, and led to enhanced socialization within the classroom. This latter aspect was confirmed during field visits, where children were seen discussing food items and their willingness to try unfamiliar foods.

*“The kids won’t always eat stuff at home, but at least they are trying it here”*

V8_Ph3

*“Getting them to a place where they need to be, where they’re available to learn”*

T1_Ph1

*“It definitely starts the day off on a good note…it settles them and gets them ready for the day”*

T3_Ph2

*“Food is social, and social is part of learning”*

AS3_Ph2

*“[I hear] kids talking [mimicking voices] “Have you tried this before?” “No.” Then the other kid would say, “I love it”, so then they’ll try it too, just because”*

T2_Ph2

With respect to socialization, a few participants also expressed that the OSNP provided additional opportunities to build relationships with their students while also providing opportunities for non-school program volunteers (e.g., parents and caregivers) to become part of the school community.

*“Meets the needs of building a relationship with the student, ‘cause they see that this is a safe place and sort of a place that cares for them…it fosters the education”*

T1_Ph1

*“Everyone enjoys the parents coming in. It kind of feels like a family. Or their kids go here and they want to feel part of the school. I love that”*

V3_Ph3

Finally, a few participants stated that the OSNP presented opportunities for enhanced learning, such as promoting both good manners, nutrition, and leadership, by including children in food preparation and delivery tasks.

### 3.3. Challenges with the Program

The main challenge mentioned by most participants was the high burden placed on personnel/volunteers to implement the program. While most participants commented on the dedication of program volunteers, they also acknowledged the large responsibility put on volunteers to plan, purchase, prepare, and deliver snacks to students. Not only did participants express difficulty in finding dedicated volunteers for the program, but some also expressed that when school personnel (typically EAs) were involved, it competed with their official school duties. These findings were confirmed during field observations. The site observer (MRI) witnessed 1–2 h of preparation time and heard comments from program volunteers regarding the additional time needed to shop for food (i.e., they were shopping on evenings and weekends).

*“It’s just a little snack, but it is a lot of work to prep it and plan it”*

V1_Ph2

*“Nobody wants to take responsibility of organizing that because there’s a lot of effort and time [so] EAs come every morning early [to do that] on top of everything else. And its volunteer work. They’re stressed out, they need support”*

T6_Ph3

*“We’d have to do [grocery shopping] on our own time or after school and then I’d have to store it at my house for the weekend”*

EA6_Ph3

Another challenge mentioned by most participants was inadequate funding, which placed additional constraints on program implementation. Some participants expressed that while they tried to adhere to nutritional guidelines, funding often limited their purchases to less expensive non-perishable items (e.g., granola bars). Other participants commented that the lack of funding to support infrastructure, such as storage and preparation utensils, had an impact on food choices (e.g., purchasing non-perishable items due to lack of fridge space). This necessitated that participants either bring supplies from home, often at their own cost, or fundraise/find donations, which led to an increased need to purchase food items (especially perishable ones) more frequently. Field observations confirmed this lack of infrastructure by noting limited space for food preparation and storage.

*“I think money is the first and foremost challenge. We work the best we can, by getting donations, or bringing in our own stuff”*

EA2_Ph1

*“You still end up defaulting to more prepared foods over fresh fruits and vegetables”*

AS3_Ph2

*“I literally have to go every single day to buy the snack”*

V3_Ph3

*“What I like least is often I am out of pocket”*

T5_Ph3

While most participants felt the program was appreciated by school staff, some mentioned that the lack of clear guidance regarding the purpose of the program led to some tensions within the school. Some participants expressed that they were unsure as to the intended program recipients (i.e., those in need vs. universality), the expectations regarding how much each child should receive, the program’s role in feeding children, and the competition with mandated instructional time.

*“Some teachers just don’t do it, or they say their kids don’t need it”*

T6_Ph3

*“It’s a delicate area because we are not parents, I don’t feel we should be the ones telling them they have to eat it”*

T9_Ph3

*“We’re supposed to teach this many minutes, they don’t account for a snack in that time”*

T7_Ph3

*“I know some teachers feel that’s it not my job, and it is our job in a way, because it is in the curriculum to teach healthy eating, right?”*

T7_Ph3

*“It’s taking time away from where I need to be in my classroom”*

T6_Ph3

Although only mentioned by a few participants, some minor issues regarding the variety and quality of foods provided were mentioned (e.g., repeat items and spoilage). This was confirmed by the Weekly Logbook data, which revealed that the quality, freshness, and appearance of snacks were rated as “very good” to “excellent” by 82%, 97%, and 98% of respondents, respectively (data not shown).

### 3.4. Potential Solutions for Program Improvement and Sustainability

All participants stated the need for adequate and sustained resources to ensure program feasibility and fidelity in the future. Most participants mentioned the need for enhanced funding to support a greater variety of snacks, including some that are pre-packaged, to alleviate the burden of preparation. Many participants commented not only on the need for more dedicated staff, but also for resources to support those staff, such as educational workshops (e.g., what to do with leftovers, how to prepare snacks for large numbers of children), access to a pre-planned menu with some flexibility to choose food items, and opportunities to improve economies of scale.

*“More money is always wonderful”*

V4_Ph3

*“It’d be so much better if we had more help”*

V2_Ph2

*“Reading the ingredients, learning the first few words on the ingredient list are really important, learning how to buy in bulk for cheap when you are feeding 520 people you gotta find cheap and just organizational skills and multi tasking on how to do this”*

V4_Ph3

*“We are all doing procurement and delivery on a small scale”*

AS3_Ph2

Another suggestion mentioned by many participants was the need to clearly communicate the role of the food program within the school environment. Participants stated that if clear links to existing curriculum were made, it might help to better communicate the expectations for the program. Some participants also commented that children’s involvement in food preparation would be welcome; however, the feasibility regarding supervision would have to be considered.

*“Communication is key. Working together is key and the culture has to change a little bit”*

T6_Ph3

*“We have to do a better job at educating teachers and understanding that this is why we’re doing it”*

T10_Ph3

*“It’s [student involvement] not really built into the program, but I think administration would support it. I think its just a difficult thing to do with supervision”*

T1_Ph1

A few participants mentioned the need to improve the visibility of the program in the hopes that it might lead to better buy-in by all stakeholders, including not only school staff and parents, but also by society in general, which ultimately could lead to more funding.

*“Making it [the program] more visible in schools, would go a long way”*

AS2_Ph1

*“You have to change the mindset of the public, right? And then have the parents be understanding of the importance, so they want to fight for it…without the public’s passion behind it, politics won’t change”*

EA1_Ph1

## 4. Discussion

This study examined the perspectives and experiences of school-level volunteers involved with a SFP in Southwestern Ontario. While the values and successes of the program were overwhelmingly acknowledged by all volunteers, some challenges were discussed, along with potential solutions for future program improvement and sustainability.

The primary value of the OSNP identified by these participants was the influence they perceived it had on children’s dietary consumption and exposure to a variety of healthy, and sometimes unfamiliar, foods. These impacts of a SFP on children’s eating patterns have been well documented in the literature. Studies in the US and Europe have shown that these programs can increase the consumption of healthy foods, such as FV [20]. They have been attributed with increasing children’s exposure and willingness to try new foods [20,21]. In this regard, the OSNP has the potential to increase exposure to a variety of healthy foods and decrease neophobia, both of which are factors known to have an impact on children’s nutrition not only in the short term (e.g., help meet FV recommendations), but also in the long term (e.g., by influencing children’s eating habits later in life) [22].

In addition to the impact on eating behaviors, the universality of the OSNP was acknowledged by participants as a means of lowering the potential stigma that can often be associated with programs that simply target children from food insecure households [23]. Although food security remains an urgent public health issue in Canada, affecting one in six Canadian children under the age of 18 [24], the role of SFP to address food insecurity continues to be debated, with some reporting SFP as a viable solution [9] and others stating they are not [25]. The potential of a SFP to have an impact on food security depends on a multitude of factors, including the dose and frequency of food provision. Furthermore, given that food insecurity is primarily associated with financial constraints [25], it is unknown whether the mere provision of food through SFP, like the OSNP, can influence children’s food security, as it does not address household financial limitations. Regardless, by offering the OSNP universally, all children can benefit from access to healthy foods at least once per day. There is also the potential to influence healthy eating behaviors in the future, independent of family income [26].

Interestingly, participants identified learning opportunities for children with the OSNP (beyond healthy eating), and the potential for greater learning in the future. They commented that the sharing of food promoted socialization and dialogue with their students. Aarestup et al. (2014) reported a sense of “hygge” or “cozy” with their SFP, which represents a safe, low-key, intimate form of socialization [21]. The sharing of food can create a sense of commensality that not only supports social skill development but may also provide an opportunity for enhanced community building [21]. Community building within the school environment is a common value of SFPs [15,21,27] and can extend beyond the school itself. Interviews with food providers in the intervention arm of the present study revealed that both food wholesalers and distributors and food providers (e.g., farmers) felt that they were contributing to their communities by supporting the OSNP [28]. Furthermore, farmers also saw their involvement as a means to connect with and increase their profiles within their communities, and to increase awareness about their practices and products [21].

In addition to social skill development, participants discussed the potential of the OSNP to support food literacy education. Food literacy, by definition, goes beyond an understanding of nutrition and food skills to a greater understanding of the complexities of food and its interaction with health and the environment [29]. Therefore, focusing the OSNP on the sharing of food while including food literacy education could greatly enhance learning while building resiliency in the form of life-skill development [29]. One question that remains, however, is the feasibility and acceptability of children’s involvement in SFP implementation. Although food preparation may enhance children’s food literacy through skill development [6,21], the logistics of supervision and maintenance of food safety would need to be addressed.

Further to the above values and successes of the program, participants also viewed the OSNP as a way to support and/or care for their students—a value that is not often mentioned in the literature. Potter et al. (2011) reported that children felt cared for by snack personnel [20]. Black et al. (2020) demonstrated that children involved in the Fuel Up! program often equated food as care and preferred it when food service staff were on site preparing their lunches as compared to having prepared lunches delivered directly to the school [14]. Taken together, the OSNP provides more that just the provision of food to children and a vehicle for enhanced learning. It can also create an overall sense of community and care not only amongst the children the program serves, but also among those involved in food provision and preparation.

Despite the many values and successes of the SFP, some challenges were mentioned. Predominant among them was the burden put on snack program volunteers to plan, procure, and prepare food. Typically, universal, nationally funded programs have access to food service staff and facilities and employ centralized food procurement practices [8,20]. That model allows for better purchasing power, greater adherence to nutritional and food safety standards, and adequate funding to support human resource and infrastructure needs. To alleviate the planning and procurement burdens of this usual practice model, the OSNP recently pilot-tested a Centrally Procured School Food Program (CPSFP), which employed a central food procurement model [15]. Interviews conducted with school-level snack volunteers and food providers in that program (e.g., OSNP regional and site coordinators) stated that this new CPSFP model did alleviate many burdens placed on staff to plan and procure food; however, the existing burden of food preparation remained, and, in some instances, the volume and types of foods being delivered to the schools created new burdens. For example, some menu items required a lot of preparation (e.g., pineapples) that further stretched existing volunteer time [15]. Therefore, even if a nationally funded program using a central food procurement model is to be considered in Canada, the human resource issue would remain and would need to be addressed by providing adequate funding for staff specifically hired to service the program, rather than relying on volunteers.

Another challenge of the OSNP was that it often contributed to competition among school priorities for school staff. For those school staff directly involved with the program (mostly EAs), the time needed to prepare snacks was shown to impede upon their existing contractual responsibilities (e.g., student supervision). For teachers, the inclusion of food into the classroom can pose different challenges, such as disruption of learning time and trepidation regarding the role of a teacher vs. the parent in feeding students. This tension between competing school priorities is a common phenomenon discussed in SFP literature [4,20,21,27]. The inclusion of a SFP into mandated school curriculum will inevitably signal its priority to school staff and would help to communicate a clear purpose for the SFP, thereby eliminating any confusion as to whom the program serves. In Ontario, Bill 216—the Food Literacy for Students Act—is currently being considered by the legislature [30]. If approved, the Act would require schools to offer experiential food literacy education to students and may provide support for the role of a SFP like the OSNP in achieving student learning outcomes.

Finally, and central to addressing many of the challenges presented with the OSNP, was the need for sustained and adequate funding. Funding is a common facilitator and/or barrier mentioned in school snack literature [12,27,31]. Not only does appropriate funding ensure that all children are reached with an adequate level of food to have an impact on their diets (e.g., a serving of FV), but also that the foods provided meet nutritional recommendations (e.g., more minimally/unprocessed vs. ultra-processed foods). Additionally, many of the burdens on snack personnel could be resolved if adequate physical and human resources were available. While an investment in a centralized food procurement model would eliminate the burden on OSNP volunteers to procure food, additional investments in storage, adequate preparation areas and utensils, and snack personnel would still be required [27].

The strengths of this study include a variety of data sources and methods of data collection. Multiple researchers also conducted data analysis, enhancing credibility. The limitations of this study include the fact that the study investigated volunteer-led snack programs in only one region of the country; therefore, the results may not be transferable to other school snack programs.

## 5. Conclusions

The findings of the present study confirm that SFP offered in OSNP offer many benefits to children. Participants provided many in-depth insights into the potential values and successes of the OSNP that extend beyond providing children with the provision of healthy foods. These include the potential of these programs to influence children’s learning, engender a sense of community belonging, and enhance life skill development. Despite its successes and the dedication of school-level volunteers to deliver the OSNP, the sustainability of this program is uncertain due to its many challenges. These challenges are mainly attributed to inadequate resources, which lead to inconsistencies in program implementation and possible inefficiencies (e.g., higher food costs). If a universal, nationally funded SFP is to be considered, policy makers and practitioners will need to assess the political will to invest in such programs. Adequate and sustained funding will be needed to ensure that schools have the proper physical infrastructure and human resources to deliver a SFP that provides appealing and healthy foods to children at the appropriate level to have any impact on their dietary behaviors. This study also demonstrates that an evaluation of best practices needs to be conducted to assess the parameters of a nationally funded program (e.g., the amount of leeway each school will be granted, if any, to implement a program based on their needs and capacities). In conclusion, SFP represent a means by which multiple sectors—public health, government, education, and the private sector—can intersect to not only meet the basic needs, education, and life skill development of children, but also to have holistic benefits in the community by supporting the local food economy, building community beyond the school environment, and providing potential employment opportunities.

## Figures and Tables

**Table 1:** Interview guide for food preparers.

What do you think about having a school food nutrition program in your school? Can you tell us your expectations for the program? Have your expectations been met? If so, how? Can you describe the challenges (if any) associated with the implementation of the program in your school? Can you describe what aspects of the program were successful and unsuccessful (if any) and why? Can you tell us what you liked most and least about the program and why? From your perspective, how is the program received by the students? From your perspective, how has the program impacted the students? Can you comment on whether or not parents are aware of your school food program? And if so, how? Have you received any feedback (e.g., from teachers, students, parents, or others) about the program? What changes or suggestions (if any) would you make about the program moving forward? Is there anything else you would like to say about the program or to the research team at Western University?

**Table 2:** School characteristics.

School Characteristics	A	B	C	D	E	F	G
Phase of implementation	I	II	II	III	III	III	III
Urbanicity	Suburban	Rural	Suburban	Rural	Suburban	Urban	Rural
School enrollment	630	130	225	520	803	900	357
School Schedule	BSD	TSD	TSD	TSD	BSD	BSD	TSD
Snack Time	Morning	Morning	Morning	Morning	Morning	Morning	Morning
Other food program	NA	NA	Milk Program	Milk and Breakfast program	NA	Milk Program	Milk program

I-Winter 2017; II-Fall 2018; and III-Winter 2018. BSD = balanced school day (2, 40-min lunch periods) and TSD = traditional school day (2, 15-min recesses and a 1-h lunch period). NA = Not applicable.

## Data Availability

Not applicable.
